# Repeat stereotactic radiosurgery in the management of brain metastases from NSCLC: A case report and review of the literature

**DOI:** 10.3892/ol.2013.1509

**Published:** 2013-08-02

**Authors:** GIULIA MARVASO, AGNESE BARONE, CATERINA VACCARO, VICENTE BRUZZANITI, SILVIA GRESPI, VALERIO SCOTTI, CATALDO BIANCO

**Affiliations:** 1 Medical Radiation Oncology Unit, Magna Graecia University and Cancer Center, Germaneto, Catanzaro I-88100, Italy; 2 Laboratory of Medical Physics and Expert Systems, Regina Elena National Cancer Institute, Rome I-00199, Italy; 3 Malzoni Radiosurgery Center, Neuromed Group, Agropoli, Salerno I-84043, Italy

**Keywords:** repeat stereotactic radiosugery, brain metastasis, neurocognitive function

## Abstract

The aims of radiotherapeutic treatment of brain metastases include maintaining neurocognitive function and improvement of survival. Based on these premises, we present a case report in which the role of repeat stereotactic radiosurgery (SRS) was investigated in a patient with a recurrent brain metastasis from non-small cell lung cancer in the same area as previously treated with radiosurgery. A 40-year-old male caucasian patient was diagnosed with brain metastasis from non-small cell lung cancer (NSCLC) and underwent SRS. The patient developed a recurrence of the disease and a second SRS on the same area was performed. After 8 months, tumor restaging demonstrated a lesion compatible with a recurrence and the patient underwent surgery. Histological diagnosis following surgery revealed only the occurrence of radionecrosis. Radiotherapy was well-tolerated and no grade 3/4 neurological toxicity occurred. To date, no consensus exists on the efficacy of retreatment with SRS. Despite the limited number of studies in this field, in the present case report, we outline the outcomes of this unconventional approach.

## Introduction

Non-small cell lung cancer (NSCLC) is the leading cause of mortality from cancer and the most common source of brain metastases. It is estimated that between 30 and 50% of lung cancer patients develop metastatic cancer to the brain during the course of their illness (1). Patients with untreated brain metastases have a median survival time of ~4 weeks and almost all succumb to neurological rather than systemic causes (2). Management and treatment of metastatic brain tumors are complex and dependent on several factors, including age, performance status, number of metastases at presentation and status of systemic disease. Radiation therapy options for brain metastases include whole brain radiotherapy (WBRT) and stereotactic radiosurgery (SRS). For a number of years, WBRT has been the primary brain metastases treatment; however, a number of studies have reported an unfavorable effect on neurocognitive function which, in turn, correlates with decreased quality of life (3,4). By contrast, SRS alone provides great benefits in managing brain metastases, representing a minimally invasive treatment with an excellent local control rate, correlating with a minor risk of neurological dysfunction (5).

Repeat SRS for recurrent metastases is a noteworthy option, although, few clinical studies support normal brain tissue tolerance to re-irradiation (6,7).

In the current case report, we present a patient with brain metastasis of NSCLC who underwent repeat SRS, analyzing the outcomes (response, survival and toxicity) and the clinical utility, and comparing our approach with other experiences described in the literature.

## Case report

### Clinical presentation and treatment

**Figure 1:**
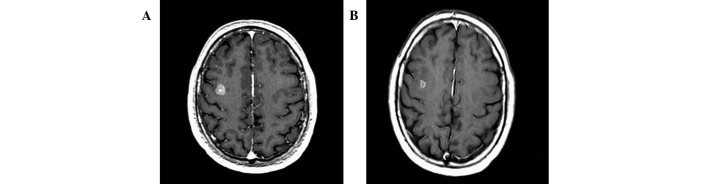
MRI of the brain. Right frontal lesion prior to (A) first and (B) second stereotactic radiosurgery treatment.

In April 2010, a 40-year-old male, with a history of NSCLC (histology, adenocarcinoma) treated from 2008, was referred to the Department of Radiation Oncology, Tommaso Campanella Cancer Center (Catanzaro, Italy) for a lesion in the right frontal lobe measuring 1.4 cm in maximum diameter (Fig. 1A). Based on RTOG criteria, the patient was eligible for SRS: Lesion measuring <3 cm in maximum diameter, producing minimal mass effect and ECOG performance status, 0.

In May 2010, the patient underwent a stereotactic treatment delivered in a single dose of 24 Gy using a Clinac DBX 600 (Varian Medical Systems Inc., Palo Alto, CA, USA) and was followed up clinically by serial MRI and CT scans. In September 2010, a complete response to the radiotherapy treatment was documented by an MRI of the brain.

### Disease recurrence

**Figure 2:**
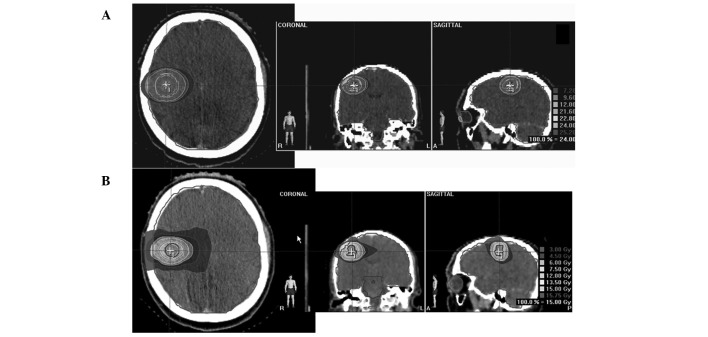
Dose distribution with 3D-CRT plan to the brain lesion. Radiosurgery plan showing the tumor volume, and (A) 24 Gy (first treatment) and (B) 15 Gy (second treatment) isodose lines covering the whole tumor.

A CT scan revealed a recurrent disease, in the same area as treated previously, 17 months after the irradiation of the metastatic brain tumor (Fig. 1B). At this point, the patient was offered a second SRS treatment as an alternative to surgery. Following obtaining consent for re-irradiation, in November 2011, the patient underwent a second session of radiosurgery, which was delivered in the same manner as the initial session, but at a single dose of 15 Gy (Fig. 2A and B). In this case, the dose was selected according to a previous study using the linear quadratic model to derive information on the cumulative biological effective tolerance dose (BED_cumulative_) that results from BED_initial_ plus BED_re-irradiation_(8).

A stable disease, associated with no symptoms or signs associated with neurological decline, was documented in the following CT scan and MRI brain controls until July 2012, when a MRI brain scan again showed a lesion measuring 1 cm in diameter in the same region and surrounded by an extensive area of vasogenic edema, compatible with a recurrence of disease.

**Figure 3:**
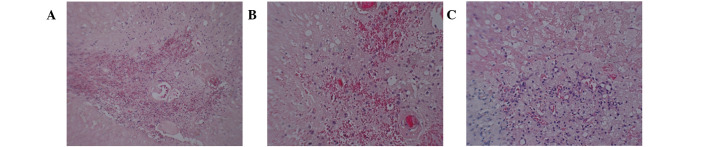
Histological examination following surgery. Pathological specificity of late cerebral radionecrosis with typical coagulation necrosis showed profound vascular changes (A), including fibrinoid necrosis and hyalinization of the wall, occlusion of lumina by fibrin thrombi with exudation of fibrinous material and hyaline material (B), and poorly active inflammatory areas with a number of inflammatory ghost cells (C). H&E staining; magnification, ×20.

Therefore, in August 2012, the patient underwent surgery to remove the right frontal lesion. Histological diagnosis following surgery showed absence of disease and the occurrence of radionecrosis (Fig. 3A–C).

At present, the patient is receiving treatment with erlotinib and appears to be in good general condition (ECOG performance status, 0).

## Discussion

When we decided to repeat SRS in the current case, it was important to scrutinize the existing evidence available regarding the use of SRS for the treatment of recurrent/progressive brain metastases. In this case, recurrent brain metastasis was defined as metastasis that has recurred at the original site of the brain following initial therapy.

The present patient belongs to category of individuals who survive long enough to experience recurrence of previously treated brain metastases but for whom no consensus on treatment exists, as the majority of studies have focused on newly diagnosed patients. In addition, few studies have demonstrated the clinical utility of repeat SRS for brain metastases.

Ammirati *et al* previously reported that there is insufficient evidence with regard to repeat SRS to generate definitive treatment recommendations in patients with recurrent/progressive brain metastases. Therefore, the treatment must be individualized based on a patient’s functional status, extent of disease, volume/number of metastases, recurrence or progression at the original site, previous treatment and type of primary cancer (9).

The value of SRS for recurrent brain metastases remains unclear and only five case studies (with Class III evidence according to the American Association of Neurological Surgeons/Congress of Neurological Surgeons criteria) (9) reported in the literature have demonstrated the clinical utility of repeat SRS and have evaluated its efficacy in patients whose previous treatment protocol included radiosurgery. In addition, only three of these case studies provide survival data from the date of SRS for recurrent disease. Yamanaka *et al*(10) and Shuto *et al*(11) reported median survival times from initial SRS of 15 and 22.4 months, respectively, in patients who repeated SRS for recurrent or newly developed brain metastases from various types of malignancies.

In a previous study by Chen *et al*, median survival from the time of SRS for recurrent brain metastases was 7 months in 45 patients (12). In addition, in a study by Kwon *et al*, 43 patients underwent salvage SRS and the median survival from the time of SRS for recurrent/progressive disease was 8 months (13). More recently, Mariya *et al* reported that repeat SRS is an effective treatment option, leading to a long survival with a decreased neurological decline (14,15). The authors analyzed 28 patients who underwent salvage radiosurgery for recurrent brain metastases from NSCLC showing a median survival from initial SRS of 26 months and 11 months for post-repeat SRS.

In our experience, based on these previous studies, repeat SRS was evaluated as the most effective alternative therapy. In addition, our patient was included in the most favorable prognostic group (baseline characteristics: age, <50 years old; KPS 90–100; 1 intracranial metastasis present and absence of extracranial metastases) for the existing criteria of a prognostic classification called Graded Prognostic Assessment, based on a previous review of the RTOG database (16).

An additional advantage of repeat SRS is the possibility to administer precisely directed, high-dose irradiation that tightly conforms to an intracranial target, in order to create a desired radiobiological response while minimizing radiation dose to surrounding normal tissue. Our primary end-point was to preserve the neurocognitive functions of the patient, considering the high risk of neurological decline with conventional radiotherapy techniques (3,4).

With regard to neurological complications of repeat SRS, Bhatnagar *et al* reported that repeat SRS was performed with minimal CNS toxicity compared with the baseline in 26 patients with benign and malignant tumors (17). In the final report of RTOG protocol 90–05, Shaw *et al* revealed the feasibility of SRS as retreatment of recurrent primary and metastatic brain tumors previously irradiated. The authors also showed that the maximally tolerated SRS dose ranged between 15 and 24 Gy, depending on the tumor size (18).

In the present case study, progression-free survival was 18 months starting from the initial radiosurgical treatment and 8 months from post-repeat SRS. The retreatment was well tolerated by the patient and no acute toxicity was observed. Neurological status was estimated from medical records, according to the criteria by Bhatnagar *et al,* based on the serial changes of 3 neurological symptoms: Seizures, focal deficits and headaches (17). None of these or any new symptoms were documented following repeat SRS. In addition, at each clinical control, neurological status and the severity of complications were rated according to RTOG CNS toxicity criteria as grade 0, according to the RTOG/EORTC scoring schema (19).

In addition, clinical chronic use of steroids was not necessary. Mini-mental state examinations were performed prior to and soon following the first and second radiotherapy treatment, and every 3 months during the follow-up, identifying a stable score ranging between 26 and 30. Repeat SRS was performed to improve local brain tumor control and reduce the neurological decline risk, enabling the patient to return to work.

Observations of the current and previous case studies discussed indicate the clinical usefulness and feasibility of repeat SRS as salvage treatment for the management of brain metastases from NSCLC, and may be a valid approach for a selected group of patients with recurrent brain metastases.
